# 79 Psychosocial Screener Outcomes for Adults in an Outpatient Burn Clinic

**DOI:** 10.1093/jbcr/irad045.053

**Published:** 2023-05-15

**Authors:** Alivia Frazier, Jamey Brumbaugh, Ariel Aballay, Jason Bregg, Christina Duncan

**Affiliations:** West Penn Hospital, Morgantown, West Virginia; West Virginia University, Pittsburgh, Pennsylvania; West Penn Hospital, Pittsburgh, Pennsylvania; West Penn Hospital, Pittsburgh, Pennsylvania; West Virginia University, Morgantown, West Virginia; West Penn Hospital, Morgantown, West Virginia; West Virginia University, Pittsburgh, Pennsylvania; West Penn Hospital, Pittsburgh, Pennsylvania; West Penn Hospital, Pittsburgh, Pennsylvania; West Virginia University, Morgantown, West Virginia; West Penn Hospital, Morgantown, West Virginia; West Virginia University, Pittsburgh, Pennsylvania; West Penn Hospital, Pittsburgh, Pennsylvania; West Penn Hospital, Pittsburgh, Pennsylvania; West Virginia University, Morgantown, West Virginia; West Penn Hospital, Morgantown, West Virginia; West Virginia University, Pittsburgh, Pennsylvania; West Penn Hospital, Pittsburgh, Pennsylvania; West Penn Hospital, Pittsburgh, Pennsylvania; West Virginia University, Morgantown, West Virginia; West Penn Hospital, Morgantown, West Virginia; West Virginia University, Pittsburgh, Pennsylvania; West Penn Hospital, Pittsburgh, Pennsylvania; West Penn Hospital, Pittsburgh, Pennsylvania; West Virginia University, Morgantown, West Virginia

## Abstract

**Introduction:**

Burn survivors are at greater risk for mental health issues, which can persist after the acute phase of injury. Many burn centers use psychological screeners for individuals receiving burn care, though few studies have reported on these results. Our study reports on the results of a routine questionnaire to screen for symptoms of depression, anxiety, PTSD, substance abuse, and suicidality in an outpatient burn clinic.

**Methods:**

We screened 669 adult patients who were receiving care at a burn clinic from October 2016 to January 2022. Each patient completed a questionnaire comprised of validated, brief measures for depression, anxiety, PTSD, alcohol use, and substance abuse. Based on scoring cutoffs, each patient’s overall score was categorized as positive/high risk (“A”), positive/moderate risk (“B”), or negative/low risk (“C”). A binary logistic regression analysis was conducted to examine potential demographic (e.g., age, gender, race/ethnicity) and injury-related predictors (e.g., TBSA, burn severity) in relation to patient screener outcomes (positive vs. negative).

**Results:**

Patients were aged 18-91 (M = 40.4; SD = 15.7) with the majority being male (N = 408; 60.6%). The mean for TBSA was 2.60 (SD=4.31) and 33.4% of the sample had 3rd to 4th degree burns. Patients were mostly non-Hispanic White (N = 572; 85.5%) and 11.2% (N = 75) were Black/African American. Most patients screened negative/low risk for psychosocial concerns (N = 502; 75%). Almost a quarter (N = 157; 23.5%) of patients screened positive/moderate risk and less than 2% (N = 10) were positive/high risk. Positive screeners were flagged most often for anxiety (i.e., occurring for 12.4% of sample). Patients also had positive screeners for depression (8.2%), PTSD (7.7%), alcohol and substance abuse (2.3%), and suicidality (1.6%). Female (OR = 1.93; p < .001) or Black/African American (OR = 2.11; p =.02) burn patients were significantly more likely to have a positive score compared to male or White patients. Burn severity significantly predicted screener outcome (OR = 1.51; p = .033; i.e., greater depth was positively related to increased risk for positive screener), whereas TBSA did not predict screener outcome.

**Conclusions:**

These findings suggest that burn care providers should be sensitive to increased risk for psychosocial difficulties in female and Black/African American burn survivors, as well as patients who sustained a more severe (i.e., depth) burn injury. Experiencing chronic racial and gender discrimination may be pre-existing factors that negatively impact psychological well-being following a burn injury.

**Applicability of Research to Practice:**

Our results highlight the importance of burn care centers incorporating routine procedures to screen for psychosocial concerns in all patients. Practitioners should also be aware of certain risk factors (e.g., burn depth; female or Black/African American patients) for poorer mental health outcomes to provide the most comprehensive care.

